# The Gut Microbiome in Hepatocellular Carcinoma: Proliferation, Inhibition, Diagnosis, and Immunotherapy

**DOI:** 10.4014/jmb.2412.12075

**Published:** 2025-05-15

**Authors:** Moo-Seung Lee, Mi-Young Son, Hyun-Soo Cho

**Affiliations:** 1 Korea Research Institute of Bioscience and Biotechnology, Daejeon 34141, Republic of Korea; 2 Korea University of Science and Technology, Daejeon 34113, Republic of Korea; 3 Department of Biological Science, Sungkyunkwan University, Suwon 16419, Republic of Korea

**Keywords:** HCC, gut microbiota, metabolite, diagnosis, immune therapy, proliferation

## Abstract

Hepatocellular carcinoma (HCC) is the third leading cause of cancer-related death worldwide. Major causes of HCC include hepatitis B and C viral infections, alcoholic hepatitis, and liver cirrhosis. Additionally, conditions such as obesity, diabetes, and metabolic syndrome have been identified as contributing factors to HCC development. In recent years, research on gut microbiota has expanded significantly, resulting in numerous studies exploring the relationship between HCC and gut microbiota. Thus, in this review, we highlight the association between gut microbiota and HCC, focusing on microbiota-related proliferation, inhibition, diagnosis, and immunotherapy. The gut microbiota is proposed to play a crucial role in both the diagnosis and treatment of HCC, paving the way for the development of novel diagnostic and therapeutic approaches for this disease.

Hepatocellular carcinoma (HCC) is one of the leading causes of cancer-related death, with its incidence increasing due to various factors, such as hepatitis B and C viral infections [1]. For HCC diagnosis, blood-based detection of alpha-fetoprotein (AFP) levels is commonly used; however, this method lacks sufficient accuracy, necessitating the development of new diagnostic approaches [2]. Although methods utilizing exosomes for diagnosis are being developed [3], a new method for diagnosing HCC is increasingly needed to establish more precise diagnostic techniques for HCC.

Therapies for HCC commonly involve the use of chemotherapeutic agents such as doxorubicin and cisplatin [4]. Additionally, for targeted therapy, drugs such as sorafenib, lenvatinib, and regorafenib have been employed. In addition, significant progress has been made in the development of immunotherapy drugs that target the programmed death 1 (PD-1)/programmed death ligand 1 (PD-L1) and cytotoxic T-lymphocyte antigen 4 (CTLA-4) pathways [5]. However, owing to low responsiveness and side effects, the demand for new therapeutic agents for HCC has been steadily increasing. Recently, the gut microbiota has emerged as a critical element that could address current limitations in HCC diagnosis and therapy. Studies have shown that metabolites produced by the gut microbiota and effective microbiota can either inhibit or promote HCC growth, increasing interest in this field [6]. Furthermore, analyses of fecal samples from HCC patients and healthy individuals have revealed the potential role of gut microbiota composition in the diagnosis of HCC [7, 8].

Thus, in this review, we highlight the significant associations between gut microbiota composition and the regulation of HCC growth, diagnosis, and responsiveness to immunotherapy. These findings underscore the vital role of the gut microbiota in the treatment and diagnosis of HCC, paving the way for innovative approaches to combat this disease.

## Diagnosis and Biomarkers of Microbiota in HCC

It has become possible to analyze the gut microbiota of HCC patients, enabling the proposal of new diagnostic methods for HCC with the rapid advancement of next-generation sequencing (NGS) technology. 16S rRNA sequencing of 292 stool samples from 124 patients with hepatitis B virus (HBV)-associated HCC, HBV-related hepatitis, and healthy controls revealed that genera such as *Dialister*, *Veillonella*, *Lactobacillus*, and species such as *Bifidobacterium faecale* and *Streptococcus pneumoniae* were associated with early recurrence of HCC [8]. Additionally, stool sample analysis of 227 patients confirmed that *Veillonella* presence is significantly correlated with increased HCC risk [9]. Metabolites produced by the gut microbiota also demonstrate associations with HCC. For instance, stercobilin and aflatoxin B1 dialcohol were found to be reduced in HCC patients, whereas triterpenoid and bafilomycin A1 levels increased. Furthermore, the *Candida* genus predominates in HCC patients [10]. Analysis of fecal matter from primary liver cancer (PLC) patients, including those with intrahepatic cholangiocarcinoma (iCCA) and HCC, revealed differences in the gut microbiota. Patients with iCCA presented a greater abundance of the *Veillonella* genus, whereas the *Blautia* genus was more prevalent in HCC patients, which suggests a potential for gut microbiome-based biomarkers to differentiate iCCA from HCC [11]. Correlation analysis of the intestinal flora in HCC patients revealed the potential diagnostic value of microbes such as *Coriobacterium*, *Atopobium*, *Coprococcus*, and *Veillonella dispar* [12]. In elderly HCC patients, an increase in *Escherichia-Shigella*, *Fusobacterium*, *Megasphaera*, and *Veillonella* abundance was observed compared with those in healthy controls. In particular, *Megasphaera*, *Prevotella* 2, and *Escherichia-Shigella* prevalence was positively associated with AFP levels [13]. Analysis of fecal samples from 20 advanced HCC patients and healthy controls showed that the levels of genera such as *Lactobacillus*, *Anaerostipes*, *Fusicatenibacter*, *Bifidobacterium*, and *Faecalibacterium* were correlated with AFP, alanine aminotransferase (ALT), and aspartate aminotransferase (AST) levels [14]. Other reports noted the enrichment of *Malassezia*, *Malassezia* sp., *Candida*, and *C. albicans* in HCC, with *C. albicans* showing increased proportions as the disease stage advanced [15]. In metabolic dysfunction-associated steatohepatitis (MASH)-related HCC, intestinal bacterial flora analysis revealed a greater abundance of *Fusobacterium* and a lower abundance of *Butyricicoccus* and *Roseburia* [16]. A genome-wide association study (GWAS) linked 11 gut microbiota species, including the *Eubacterium hallii* group, *Candidatus* *Soleaferrea*, and *Victivallales*, to HCC and biliary tract cancer (BTC) risk [17]. This study also identified *Clostridium leptum* as a protective factor, with its increased abundance reducing HCC risk by 38% and phosphoethanolamine (PE) levels by 9% [18]. Post-operative prognosis prediction in HCC has now become possible, with Proteobacteria and Actinobacteria frequently detected in the tumor microenvironment (TME). Microbial profiles within the TME vary by hepatotype, serving as independent prognostic factors for HCC [19].

**Fig. 1:**
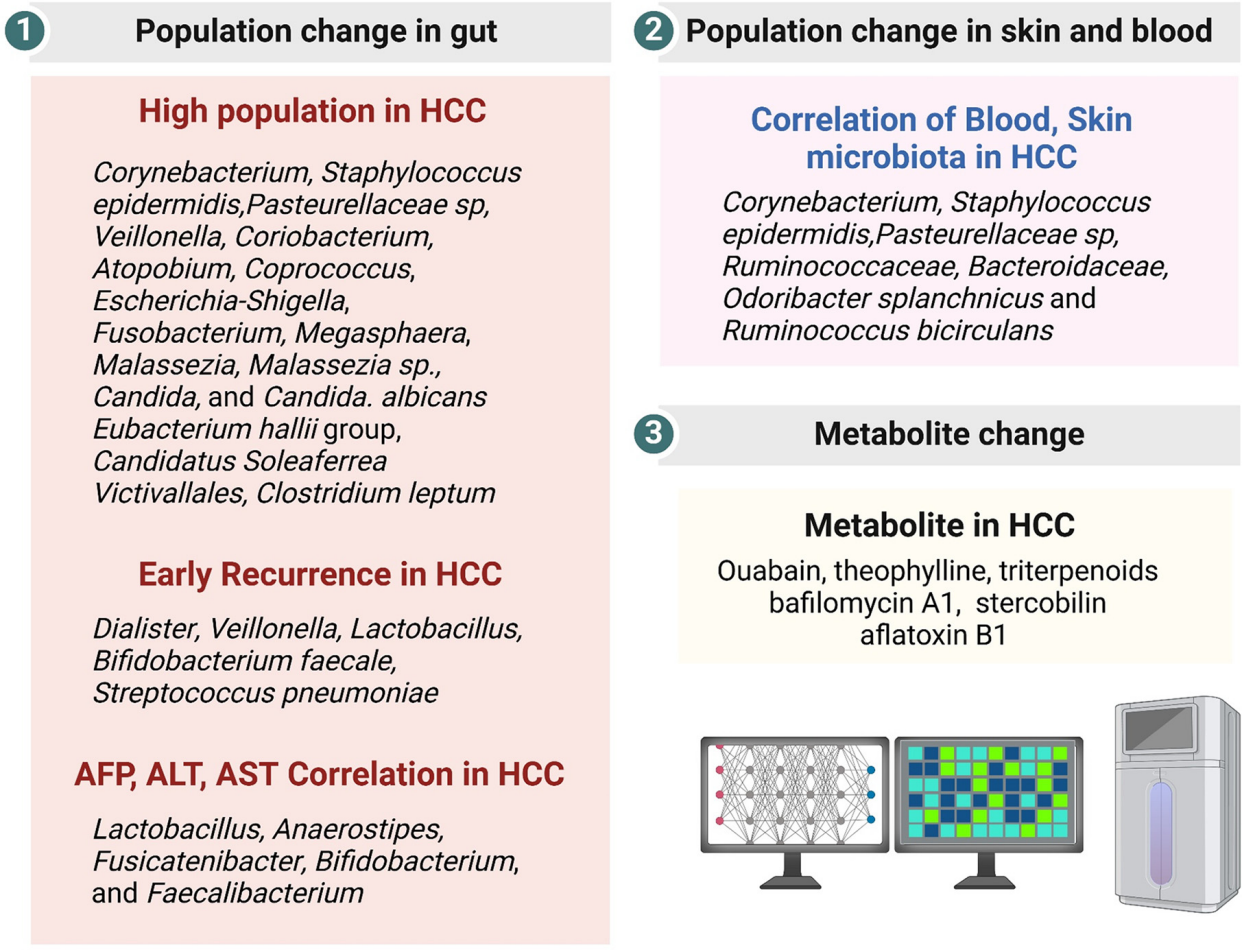
Microbiota in the diagnosis of HCC. (**1**) Diagnosis of HCC through changes to microbial communities. A correlation between microbial communities and AFP levels has been established. Changes to microbial communities associated with HCC are observed in cases of early recurrence and high risk. (**2**) Microbial communities in the blood and skin are linked to HCC. (**3**) Changes in metabolites are associated with HCC.

The correlation between the skin microbiota and HCC revealed that *Corynebacterium*, *Staphylococcus epidermidis*, and *Pasteurellaceae* sp. were associated with an increased risk of HCC. Conversely, *Betaproteobacteria* and *Firmicutes* have been linked to HCC suppression, suggesting that skin microbiota analysis could potentially aid in diagnosing HCC [20]. Moreover, urinary microbiome analysis has also been proposed as a tool for predicting HCC. A study analyzing urinary samples from 471 HCC patients and 397 healthy controls developed a urinary microbiome-derived signature for HCC [21]. Comprehensive microbiome analysis of blood, fecal matter, and liver tissue from HCC patients showed an increase in fecal bacterial gene signatures in the blood. Additionally, *Ruminococcaceae* and *Bacteroidaceae* were enriched in the blood and liver tissue of HCC patients [22]. Comparative analysis of the gut microbiota and serum metabolite profiles of patients with HCC, patients with cirrhosis, and healthy individuals identified two bacterial species (*Odoribacter splanchnicus* and *Ruminococcus bicirculans*) and five metabolites (*e.g.*, ouabain and theophylline) associated with HCC. This approach demonstrated a greater diagnostic accuracy than AFP-based methods, suggesting that these species and metabolites are potential biomarkers for HCC diagnosis [23]. In conclusion, comparisons between the gut microbiota of HCC patients and healthy controls have identified various HCC-specific microbial populations. Metabolite analysis further enhances diagnostic accuracy, indicating that the gut microbiota and its metabolites hold promise for advancing HCC diagnosis and treatment strategies (Fig. 1).

## Proliferation-Related Microbiota in HCC

Fecal microbiota analysis throughout the liver cancer progression cycle—ranging from simple fatty liver (SFL) to MASH and MASH-HCC—revealed dynamic alterations in microbial composition. At the genus level, *Bifidobacterium* and *Lactobacillus* were found to decrease as the disease advanced, whereas *Kineothrix*, *Lactococcus*, and *Akkermansia* increased, suggesting that stage-specific microbiota dynamics are closely linked to MASH progression and its transformation into HCC [23]. Mouse studies have demonstrated that maternal obesity significantly increases HCC risk in female offspring. Analysis of female offspring from obese maternal mice revealed changes in the gut microbial composition, indicating that gut microbiota transmission plays a crucial role in HCC development [24]. Additionally, comparisons between intratumor tissues and adjacent nontumor tissues showed alterations in metabolites such as *N*-acetyl-D-glucosamine, suggesting an association between various bacteria with these metabolites. These findings present the potential of targeting the microbiota and its metabolites for HCC treatment and biomarker development [25]. In HCC tumor tissue, increased abundance of *Cyanobacteria* and *Acidobacteria* was observed, while *Proteobacteria* and *Firmicutes* levels were decreased. These results suggest that the metabolic pathways of *Cyanobacteria* and *Acidobacteria* play a significant role in promoting HCC growth [26]. Conversely, the oral administration of a probiotic bacterial cocktail containing *Bifidobacterium longum* to HCC patients resulted in improved survival rates exceeding one year. The probiotic intervention reduced liver fibrosis and hepatocyte proliferation through alterations in metabolic pathways, particularly the gut microbiota-produced metabolite 5-hydroxytryptamine, which was implicated in these effects [27]. A high-fructose diet was shown to increase the amount of microbiota-derived acetate utilized by cancer cells, increasing the biosynthesis of uridine diphospho-*N*-acetylglucosamine (UDP-GlcNAc). This, in turn, promoted HCC cell growth and progression [28]. Moreover, treatment with celastrol, which is extracted from thunder god vine, reduced the abundance of *Bacteroides fragilis* and increased the levels of glycoursodeoxycholic acid (GUDCA), a bile acid. GUDCA was shown to induce cell cycle arrest by regulating the mTOR/S6K1 pathway in liver cancer cell lines, thereby suppressing their proliferation. These findings highlight the potential of natural compounds to modulate the gut microbiota and inhibit HCC growth through their metabolites [29].

**Fig. 2:**
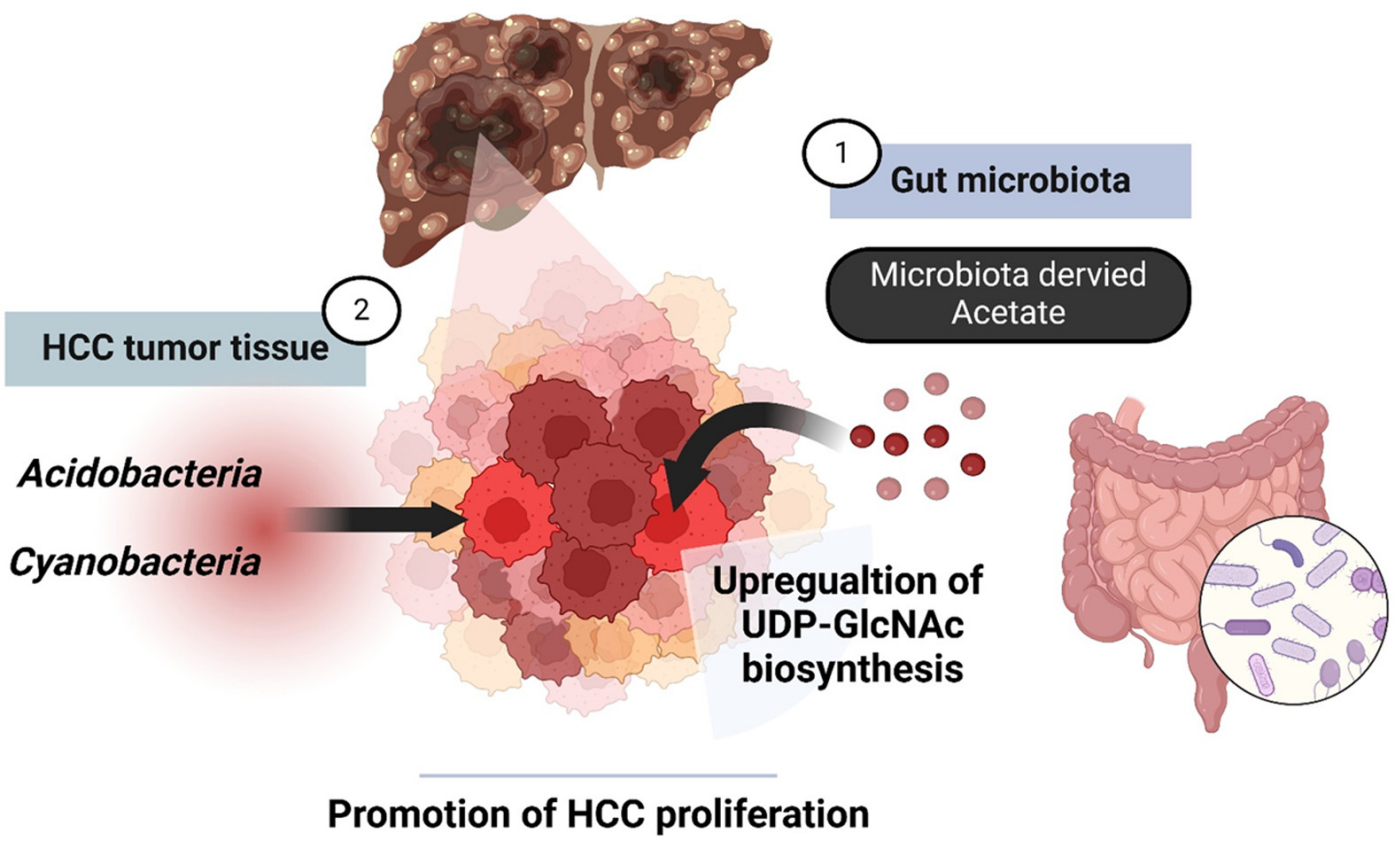
Positive regulation of HCC proliferation by microbiota and metabolites. (**1**) HCC growth can be promoted by utilizing acetate produced by the gut microbiota. (**2**) Various metabolites generated by *Acidobacteria* and *Cyanobacteria* present in HCC tissue contribute to the promotion of HCC growth. The gut microbiota and HCC-associated microbiota are closely linked to the progression of HCC.

In summary, research on the composition of microbiota and its metabolites in and around HCC tissues has revealed a strong association between microbial dynamics and HCC proliferation (Fig. 2). These findings provide insight into novel therapeutic strategies that combine microbiota modulation and anticancer agents to produce more effective treatments for HCC.

## Growth Inhibition by Microbiota in HCC

An increasing number of studies demonstrate that HCC growth can be inhibited by various metabolites and beneficial microorganisms in the gut microbiota. For example, the level of *B. longum* was found to be specifically decreased in an HCC mouse model. Treatment of the AML12 cell line with *B. longum* effectively attenuated TGF-β1-induced apoptosis and fibrosis. Furthermore, extracellular vesicles (EVs) derived from *B. longum* were shown to significantly reduce tumor formation in a diethylnitrosamine (DEN)-induced HCC mouse model [30]. The use of probiotic mixtures allows for the modulation of the gut microbiome. Studies utilizing high-fat diet (HFD)-induced MASLD and MASH murine models have demonstrated that probiotic mixtures can effectively regulate the progression from MASLD to MASH [31]. In MASLD patients, a reduction in *Adlercreutzia equolifaciens* was observed, and *in vivo* and *in vitro* models confirmed its anti-inflammatory effects via reduced interleukin-6 (IL-6) expression [32]. Additionally, in a mouse model of MASLD-HCC, the abundance of *Bifidobacterium pseudolongum* was significantly reduced. Oral gavage administration of *B. pseudolongum* inhibited MASLD-HCC formation, whereas treatment of MASLD-HCC cell lines with *B. pseudolongum* culture medium increased G1/S arrest and apoptosis. This effect was attributed to the acetate produced by *B. pseudolongum*, which inhibits the IL-6/JAK1/STAT3 signaling pathway, thereby suppressing MASLD-HCC progression [33]. Thus, these findings suggest that controlling MASLD, a risk factor for HCC, could effectively reduce HCC incidence. Another risk factor, type 2 diabetes mellitus (T2DM), was examined in a study using a T2DM + HCC mouse model, which showed that the administration of *Lactobacillus brevis* improved blood glucose levels and insulin resistance. *L. brevis* also delayed HCC progression by regulating the MMP9 and NOTCH1 signaling pathways [34]. In addition, in HCC patients, the level of butyrate, a key gut microbiota metabolite, was reduced. *In vitro* studies revealed that butyrate supplementation or depletion of butyrate metabolism-related enzymes disrupted intracellular calcium homeostasis, thereby inhibiting HCC proliferation [35].

Recent studies have reported the increased use of plant extracts and various compounds to modulate the gut microbiome for HCC inhibition. Echinacea purpurea polysaccharide (EPP) was shown to effectively inhibit HCC growth and induce apoptosis. EPP intervention increased short-chain fatty acid (SCFA)-producing gut microbiota, which regulated lipopolysaccharide (LPS) leakage and suppressed HCC growth in mice [36]. Berberine, a plant extract, was found to stimulate gut microbial metabolites that promoted PPAR-delta degradation, leading to apoptotic death of HCC cells [37]. Similarly, Ulva lactuca polysaccharide (ULP), derived from green algae extract, was shown to induce changes in gut microbial populations and metabolites, inhibiting HCC growth. ULP treatment suppressed the expression levels of JNK, c-JUN, PI3K, and Akt [38].

**Fig. 3:**
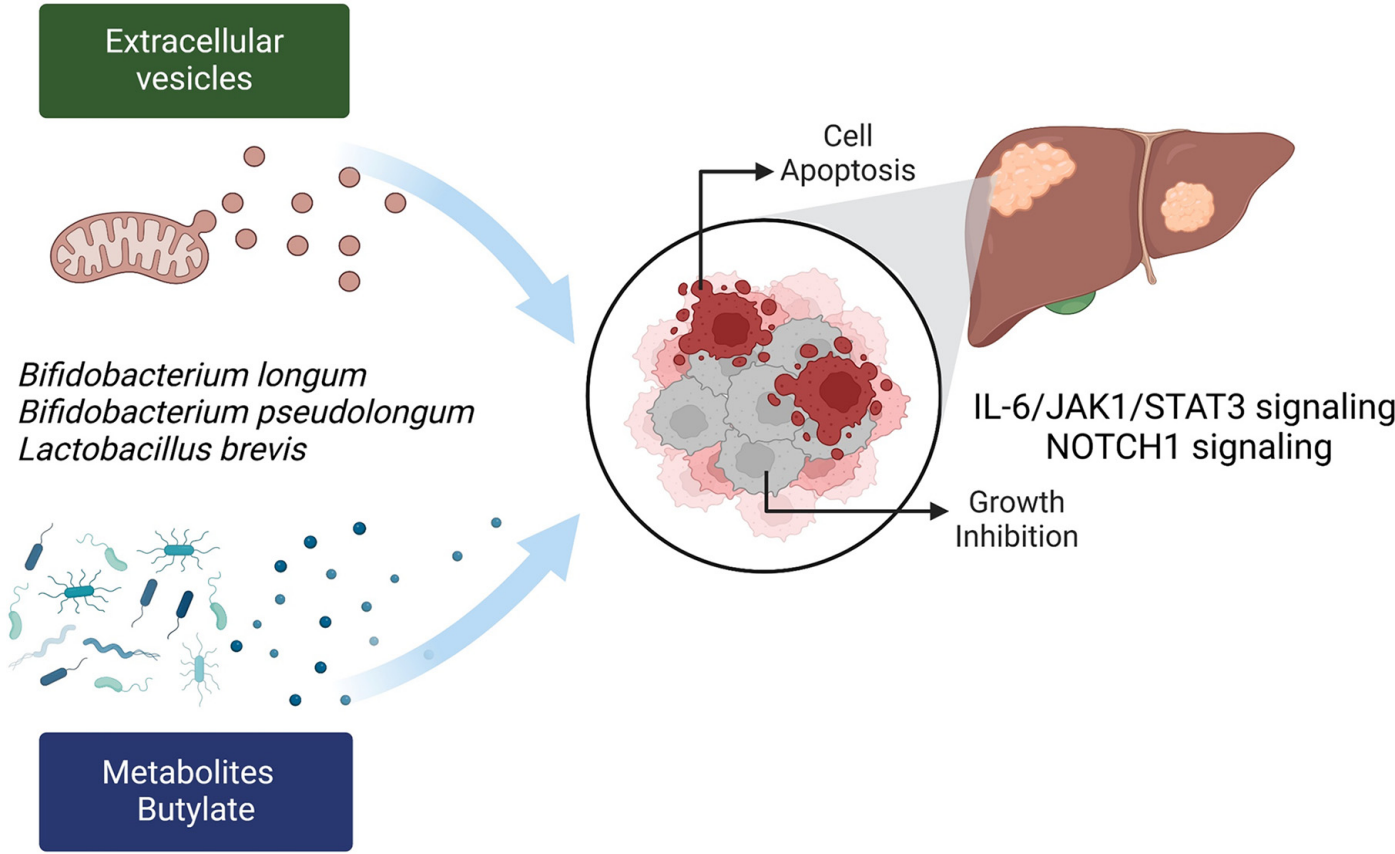
Growth inhibition of HCC by the gut microbiota and metabolites. HCC cell apoptosis is induced by the gut microbiota and its metabolites, leading to the inhibition of cell growth. Extracellular vesicles (EVs) produced by the microbiota suppress HCC growth. SCFAs, such as butyrate, induce apoptosis in HCC cells. EVs and their metabolites regulate signaling pathways, including NOTCH1 and JAK1/STAT3, to inhibit HCC growth.

Thus, the studies reviewed here indicate that changes in the gut microbiota and its metabolites can significantly inhibit HCC growth (Fig. 3). These findings highlight the potential of gut microbiota modulation as a novel strategy for HCC treatment, offering promising avenues for future therapeutic development.

## Synergistic Effects of Anticancer Drugs and the Microbiota

Various targeted therapies, such as sorafenib and regorafenib, as well as immune checkpoint inhibitors, including atezolizumab and bevacizumab, have been used to enhance the therapeutic efficacy of HCC treatment. Recently, significant progress has been made in understanding how the gut microbiota can enhance the effectiveness of anticancer drugs in the treatment of HCC. *Inukai* *et al*. analyzed fecal samples before and after the administration of atezolizumab and bevacizumab. *Bacteroides stercoris* and *Parabacteroides merdae* were more abundant in the responder group compared to the non-responder group, which was associated with poor prognosis. This finding suggested that differences in the gut microbiota composition were linked to the therapeutic effects of atezolizumab and bevacizumab [39]. Additionally, the reduced efficacy observed in HCC patients pretreated with antibiotics prior to combination therapy with atezolizumab and bevacizumab further highlights the crucial role of the gut microbiota in enhancing the effectiveness of immune checkpoint inhibitors [40]. However, other studies have reported that administering butyrate-producing enterobacteria during atezolizumab and bevacizumab combination therapy does not improve survival time or therapeutic efficacy [41]. Thus, these findings indicate the need for further research on the relationship between immune checkpoint inhibitors and the gut microbiota.

Research on targeted therapies such as sorafenib has shown that butyrate supplementation increases the therapeutic efficacy of sorafenib in the treatment of HCC [35]. Furthermore, co-administration of *Lactobacillus rhamnosus* and regorafenib in an HCC mouse model increased gut permeability and reduced systemic and intestinal inflammation. This approach decreased systemic adverse effects and reduced tumor resistance to regorafenib [42]. The combination of low-dose cisplatin with *Brassica rapa* L. polysaccharides significantly suppressed tumor growth in patients with HCC. This combination also reduced cisplatin-induced side effects, such as immune deficiency, and modulated intestinal flora dysregulation, including changes in *Lactobacillus murinus* and *Clostridiales* bacterial populations [43]. Therefore, the gut microbiota or its metabolites could have synergistic effects with anticancer drugs, potentially leading to the development of highly effective anticancer therapies with fewer side effects.

## Immune System Regulation by Microbiota in HCC

To treat HCC, immune checkpoint inhibitors PD-1 and PD-L1 have been extensively developed and are being used to treat advanced HCC. Recently, studies have increasingly suggested that the gut microbiota and metabolites are associated with the efficacy of immune checkpoint inhibitors (ICIs) in patients with HCC. Analysis of ICI-treated HCC patients indicated that *Actinomyces* sp. ICM47 and *Senegalimassilia anaerobia*, along with the metabolite galanthaminone, could serve as prognostic biomarkers for predicting survival in HCC patients receiving ICI treatment [44]. Treatment with stigmasterol, an active phytosterol compound found in plant oils and seeds, was shown to reduce tumor size in mice and increase the abundance of *Lactobacillus johnsonii*, *Lactobacillus murinus*, and *Lactobacillus reuteri*, enhancing the immune response in the HCC tumor microenvironment by increasing the number of interferon-gamma (IF*N*-γ)+ CD8+ T cells and Treg cells [45]. Additionally, acetic acid derived from *Bacteroides thetaiotaomicron* has been found to regulate the expression of acetyl-CoA carboxylase 1 (ACC1), a key enzyme in fatty acid biosynthesis. This regulation modulates the immune microenvironment, altering the polarization of proinflammatory macrophages and enhancing the function of cytotoxic CD8+ T cells, ultimately suppressing HCC tumor growth [46]. Furthermore, combination therapy with quercetin, known for its antioxidant and anti-inflammatory properties, and an anti-PD-1 antibody was found to induce changes in the gut microbiota, increasing the abundance of *Firmicutes*, *Actinobacteria*, *Akkermansia*, and *Dubosiella*. Additionally, it enhanced macrophage immunity by upregulating the expression of M2 macrophage-related genes, such as arginase-1 (Arg-1), IL-10, and transforming growth factor-β (TGF-β), while downregulating the expression of M1 macrophage-related genes, including IL-6, IL-12a, and IL-1β. These findings suggest that combination therapy using quercetin and an anti-PD-1 antibody could improve HCC treatment by regulating the gut microbiota and macrophage immunity to modulate the tumor microenvironment [47]. Tertiary lymphoid structures (TLSs) have been associated with favorable responses to immunotherapy in most solid tumors, including HCC. A comparison of intratumoral TLSs (It-TLSs) and desertic TLSs (De-TLSs) in HCC revealed a greater distribution of *Lachnoclostridium* in It-TLSs, suggesting its potential as a biomarker for predicting immunotherapy responsiveness [48]. Moreover, treatment with 2,5-dimethylcelecoxib (DMC), a microsomal prostaglandin E synthase-1 (mPGES-1) inhibitor, was found to suppress HCC growth and PD-L1 expression. *In vivo* studies further confirmed that DMC treatment regulated the gastrointestinal microbiota-AMPK-mTOR axis, increasing the expression of IF*N*-γ in NK and T cells while inhibiting PD-1 expression, ultimately leading to suppressed HCC growth [49]. Interestingly, modulation of the microbiome with antibiotics was found to influence the efficacy of ICIs. Early exposure to antibiotics is associated with poorer overall survival and progression-free survival in HCC patients, suggesting that the gut microbiota composition plays a crucial role in determining the therapeutic efficacy of ICIs [50].

## The Gut microbiota in HBV-Related HCC

Hepatitis virus infections, particularly hepatitis B virus (HBV) and hepatitis C virus (HCV), drive the pathological processes underlying virus-related liver cancer. HBV infection in particular is a major contributing factor to HCC and presents significant challenges for its treatment [51]. *Levilactobacillus brevis* SR52-2 and *Levilactobacillus delbrueckii* subsp. *bulgaricus* *Q80* have demonstrated anti-HBV efficacy by significantly suppressing the expression and levels of HBeAg. Additionally, lactic acid bacteria markedly increased total short-chain fatty acids (SCFAs) in the HCC groups, suggesting that lactic acid bacteria not only improve gastrointestinal health but also exert antiviral effects. [52]. Additionally, analysis comparing HBV-related HCC and non-HBV/non-HCV HCC identified *Cutibacterium* as a key biomarker for HBV-related HCC and revealed an association between the HBV-related HCC microbiota and increased numbers of tumor-infiltrating CD8+ T lymphocytes [53]. Fecal sample analysis of 18 HCC patients, 17 HBV-HCC patients, 16 HCV-HCC patients, and 16 healthy controls showed increases in 11 genera, including *Faecalibacterium*, *Agathobacter*, and *Coprococcus*, whereas *Bacteroides*, *Streptococcus*, and *Ruminococcus gnavus* were decreased in the HCC groups [54]. Furthermore, the analysis of HBV-HCC samples with and without microvascular invasion (MVI) suggested the potential of the gut microbiota as a noninvasive biomarker [55]. Compared with healthy samples, chronic HBV infection samples showed an increased proportion of *Clostridium* species, such as *Clostridium perfringens*, *Clostridium sporogenes*, and *Enterocloster aldenensis*. The discovery of ethanol-producing *E. bolteae* strains suggests that these strains may contribute to the progression of liver diseases such as HCC [56]. Moreover, analysis of 106 HBV-related liver disease samples (chronic hepatitis B, liver cirrhosis, and HCC) revealed an increased *Firmicutes-to-Bacteroidetes* ratio [57] compared with healthy controls. Changes in gut microbial patterns were observed in 364 HBV-HCC and 160 healthy samples, with an AUC value of 0.79710.8084, which further increased to 0.9811 when combined with AFP analysis [58]. These findings suggest that analyzing gut microbiota signatures can enable the prediction of HBV-HCC subgroups and provide insight into more effective therapeutic approaches.

## Conclusion and Outlook

**Fig. 4:**
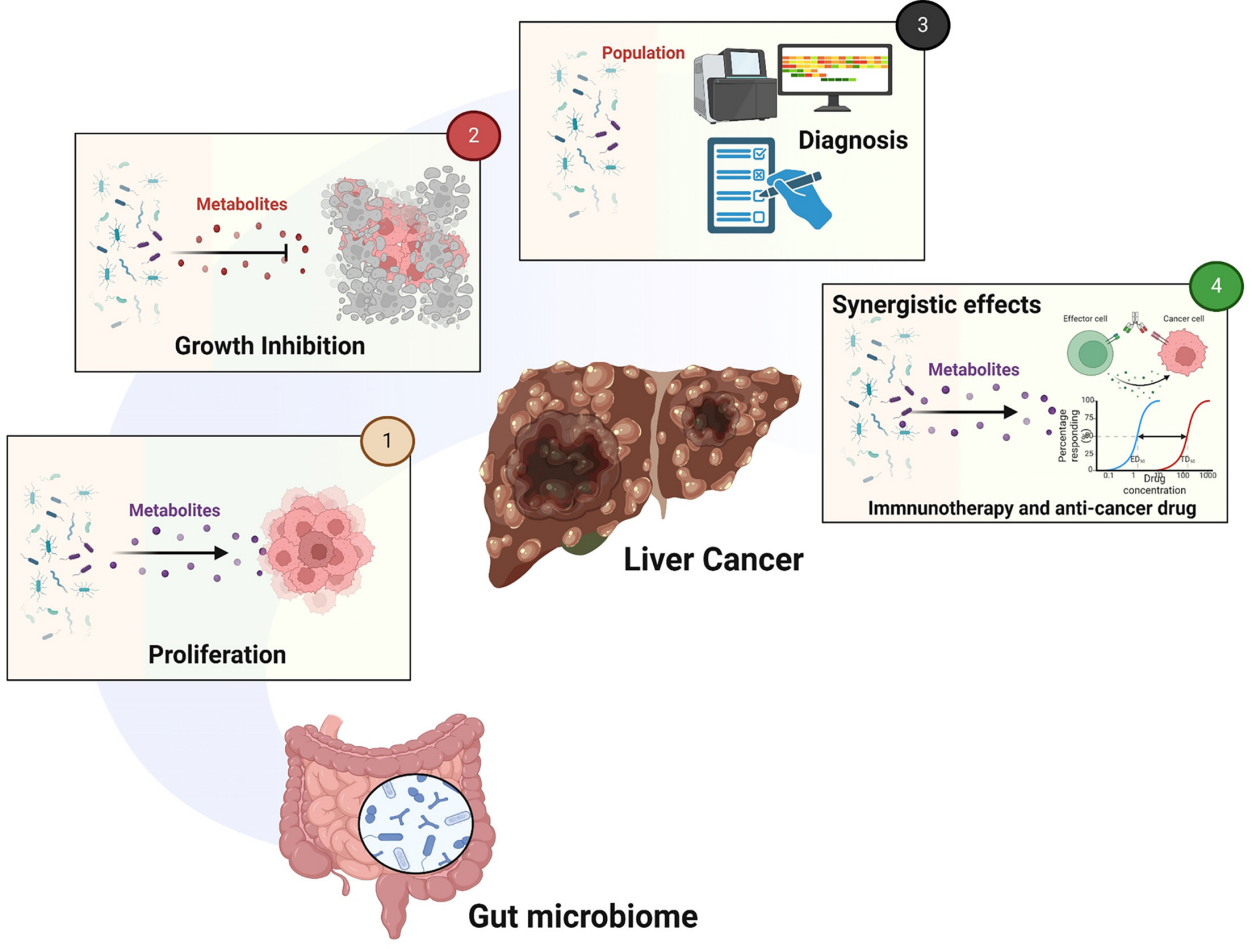
Summary of the HCC-related microbiome. Changes to the population and metabolites of the gut microbiota in HCC patients. (**1**) Various metabolites produced by the gut microbiota promote HCC growth. (**2**) Metabolites such as SCFAs inhibit HCC growth. (**3**) Changes in the population of the gut microbiota can be used for HCC diagnosis. (**4**) Gut microbiota metabolites enhance the efficacy of immunotherapy and anticancer drugs.

This review has highlighted the intricate relationship between HCC and the microbiota, demonstrating that alterations to diverse subpopulations within the gut microbiota may aid in the diagnosis of HCC. Moreover, combining microbiota analysis with AFP enhances diagnostic accuracy. This review also emphasized the pivotal role of gut microbiota composition in determining responsiveness to immune checkpoint inhibitors, underscoring its importance in the effectiveness of immunotherapy for HCC. Additionally, the use of microbiota-derived metabolites that suppress HCC and various compounds that regulate gut microbiota composition has shown potential in hindering HCC progression (Fig. 4). Despite numerous studies linking the microbiota to HCC, further research is required to elucidate the precise mechanisms by which the microbiota suppresses HCC and enhances the immune response. Identifying microbiota-derived metabolites that inhibit HCC and investigating their mode of action (MOA) will also be crucial. Thus, these findings could pave the way for the development of effective microbiota-based drugs for treating HCC. When combined with existing treatments such as anticancer drugs, immunotherapy, and targeted therapies, these microbiota-based approaches could significantly enhance therapeutic efficacy in HCC treatment.
